# Supplementary material for: Platelet aggregates detected using quantitative phase imaging associate with COVID-19 severity
Source: Commun Med (Lond). 2023 Nov 7;3:161. doi: 10.1038/s43856-023-00395-6 (PMC10630365; doi:10.1038/s43856-023-00395-6)
Supplement: Supplementary file 2 — Supplementary File [file 43856_2023_395_MOESM2_ESM.pdf]

Platelet aggregates detected using quantitative phase imaging associate with COVID-19 severity - Supplements

Christian Klenk[1#], Johanna Erber[2#], David Elias Fresacher[1,3], Stefan Röhrl[3], Manuel Lengl[3], Dominik Heim[1], Hedwig Irl[4], Martin Schlegel[4], Bernhard Haller[5], Tobias Lahmer[2], Klaus Diepold[3], Sebastian Rasch[2#], Oliver Hayden[1#*]

[1] Technical University of Munich, Heinz-Nixdorf-Chair of Biomedical Electronics, School of Computation, Information, and Technology, TranslaTUM, 81675 Munich, Germany

[2] Technical University of Munich, School of Medicine, University Hospital rechts der Isar, Department of Internal Medicine II, 81675 Munich, Germany

[3] Technical University of Munich, Chair for Data Processing, School of Computation, Information, and Technology, 80333 Munich, Germany

[4] Technical University of Munich, School of Medicine, University Hospital rechts der Isar, Department of Anaesthesiology and Intensive Care Medicine, 81675 Munich, Germany

[5] Technical University of Munich, School of Medicine, University Hospital rechts der Isar, Institute of AI and Informatics in Medicine, 81675 Munich, Germany

[#]These authors contributed equally to this work.

Corresponding author:

Prof. Dr. Oliver Hayden, MBA

Heinz-Nixdorf-Chair of Biomedical Electronics

School of Computation, Information, and Technology

TranslaTUM, Campus Klinikum rechts der Isar, Bau 522

Einsteinstraße 25, 81675 München, Germany, European Union

Phone: +49 89 41409031

Cell: +49 171 954 6 945

Mail: oliver.hayden@tum.de

Supplementary figures and tables

Supplementary Figure 1: Channel properties at measurement conditions

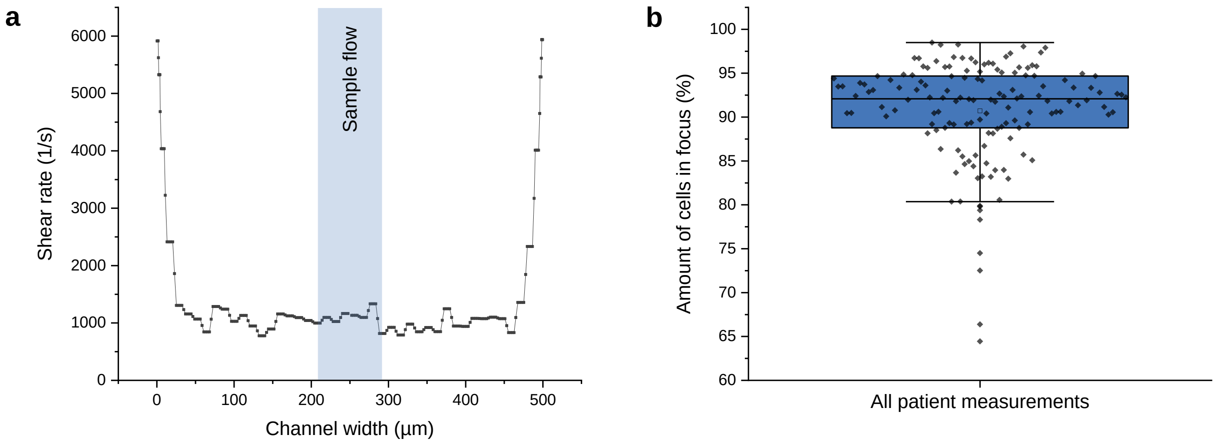

Analysis of the shear rate distribution in the microfluidic channel and the amount of focussed blood cells. **a,** To determine the shear rates during the measurement conditions, simulations were performed in COMSOL Multiphysics 5.6. The sample flow (highlighted in blue) is focused on the centre of the channel by two side sheaths, as explained in the methods section. The shear rate distribution along the horizontal axis is shown. **b,** Overview of the amount of blood cells in focus for all patient measurements. The fraction of cells in focus was analysed using the Sobel gradient operator with fixed threshold values. The distribution is visualised by a boxplot. Each dot of the graph represents the mean for one single measurement. The centreline of the boxplots represents the median, while the bottom and top borders of the box correspond to the first and third quartiles.

Supplementary Figure 2: Effect of other influencing factors on individual platelets and platelet aggregates

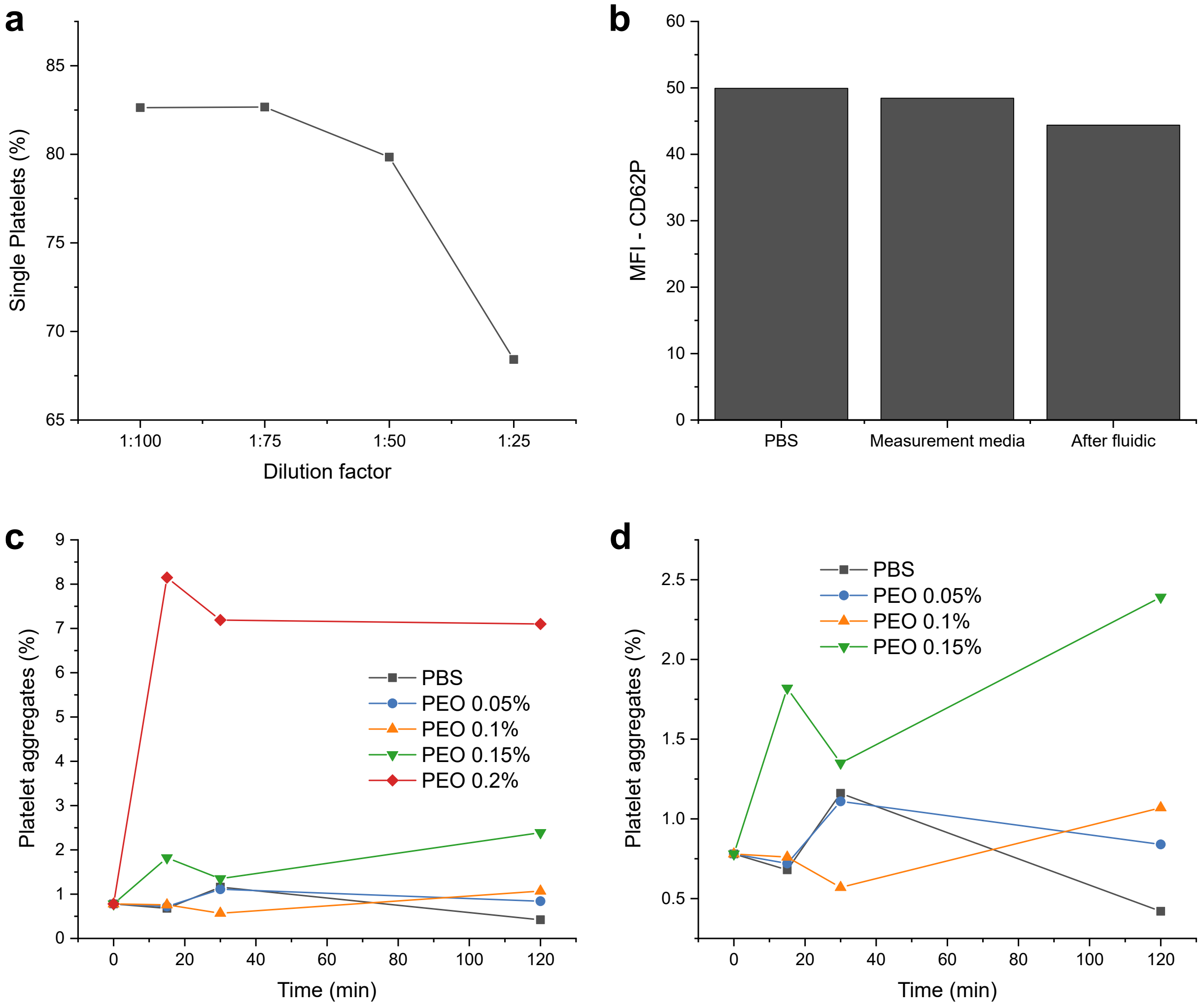

a, Measurement of single platelets of the same blood sample for four different dilution levels to determine coincidences of single platelets appearing as aggregate. With a dilution of 1:75 or higher, coincidences of single platelets are minimized. All reported measurements are performed with a dilution of 1:100. **b,** The effect of fluidics on platelet activation was assessed using the P-selectin maker CD62P. Only marginal differences between the Mean Fluorescence Intensity (MFI) of platelets in PBS, in measurement medium (0.05% PEO), and measurement medium after passing the fluidic chip can be measured. An increased MFI value is expected for an increased activation of platelets. **c-d,** Measurement of the influence of different PEO concentrations on the formation of PP aggregates. Platelets were stored at four

different concentrations of PEO for 120 minutes and compared with platelets in PBS. While comparing PBS, PEO 0.05% and PEO 0.1%, no major difference was observed. For PEO 0.15%, a slight PP aggregate increase was measured after 15 minutes. This effect was even more evident with PEO 0.2%. Here, the PP aggregates increased up to eight times the initial value.

Supplementary Figure 3: Exemplary image of a single measurement frame

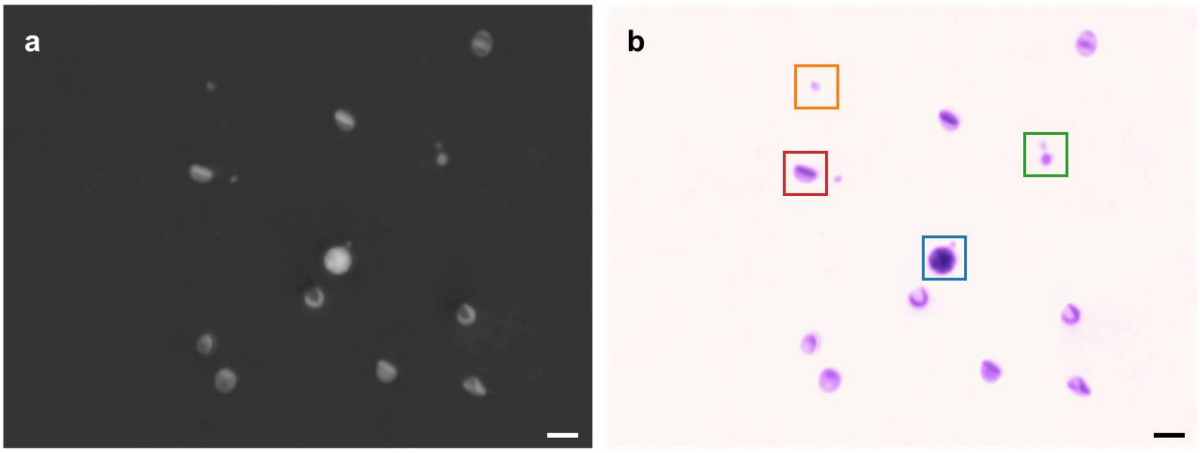

A single quantitative phase contrast image is represented in grey values (**a**) and false colours (**b**). Only the phase contrast images and no amplitude images or holograms were used to detect PP and LP aggregates. Each frame shows more than one cell, allowing a parallel measurement to achieve a high-throughput and statistical yield despite the low flow rate to minimize mechanical stress. An exemplary platelet, erythrocyte, LP aggregate, and PP aggregate are highlighted in orange, red, blue, and green, respectively. The scale bars correspond to 10 µm.

Supplementary Figure 4: Overview of measurement days and intubation duration of the patient collective

a **b**

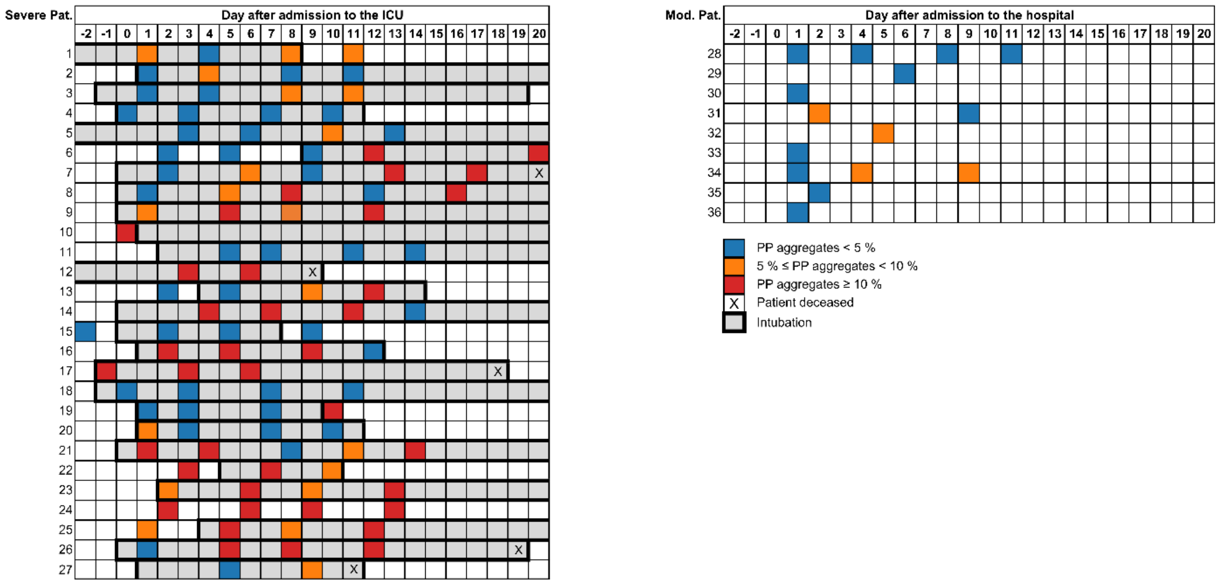

Tabular overview of patients with severe (**a**) and moderate courses (**b**). The coloured scare indicates measurement time points, with the colours representing less than five percent measured PP aggregates (blue), between five and ten percent PP aggregates (orange) and greater than ten percent PP aggregates (red). Crosses mark the day of death if this happened within 20 days of admission to the ICU. Of note, the time scale for moderate patients starts with admission to the hospital. Mechanical ventilation is highlighted in grey.

Supplementary Table 1: Baseline characteristics of the patient and healthy reference collective

Baseline characteristics of COVID-19 patients	Mean (SD) or Median (Range)	n/N (%)	missing data
Age (years)	65 (32-83)		0
Female		12/36 (33.3)	0
Male		24/36 (66.7)	0
Ethnic group<br>White		 33/36 (91.7)	0

Black<br>Other		2/36 (5.6)<br>1/36 (2.8)	
Body mass index (kg/m^2)<br>18.5-24.9<br>25-29.9<br>30-34.9<br>35-39.9<br>>40		 6/36 (16.7)<br>14/36 (38.9)<br>4/36 (11.1)<br>5/36 (13.9)<br>2/36 (5.6)	5
Smoking status<br>Non-smoker<br>Current smoker<br>Former smoker		 22/36 (61.1)<br>0/36 (0)<br>6/36 (16.7)	8
**COVID-19**			
WHO clinical progression score	9 (4-10)		0
Variants<br>Wildtype<br>B.1.617.2<br>B.1.1.7<br>B.1.351		 10/36 (27.8)<br>21/36 (58.3)<br>4/36 (11.1)<br>1/36 (2.8)	0
Known previous SARS-CoV-2 infection		0/36 (100%)	0
Doses of COVID-19 vaccine >14 days before study inclusion<br>None<br>1 dose<br>2 doses		 26/36 (72.2)<br>1/36 (2.8)<br>8/36 (22.2)	1
**Admission details**			
Length of stay in hospital (days)	26 (16.7)		0
ICU admission		8/36 (77.8)	0
Length of stay on ICU (days)	16.5		0
Death during hospitalization		13/36 (36.1)	0
**Therapeutic details**			
Oxygen (nasal cannula or mask)		33/36 (91.7)	0
Non-invasive ventilation		15/36 (41.7)	0
Mechanical ventilation		26/36 (72.2)	0
Length of mechanical ventilation (days)	18 (1-43)		1
Prone positioning		20/36 (55.6)	0
Extracorporeal CO_2 elimination		10/36 (27.8)	0
ECMO therapy		4/36 (11.1)	0

Renal replacement therapy		10/36 (27.8)	0
**COVID-19 directed therapy**			
Remdesivir		5/36 (13.9)	0
Dexamethasone		32/36 (88.9)	0
Casirivimab/Imdevimab		9/36 (25)	0
Tocilizumab		2/36 (5.6)	0
**Comorbidities**			
Arterial hypertension		20/36 (55.6)	0
Diabetes mellitus		10/36 (27.8)	0
Coronary heart disease		6/36 (16.7)	0
Congestive heart failure		4/36 (11.1)	0
COPD		2/36 (5.6)	0
Bronchial asthma		1/36 (2.8)	0
Malignant disease		5/36 (13.9)	
Chronic kidney disease		6/36 (16.7)	
**Baseline characteristics of healthy donors**	**Mean (SD) or Median (Range)**	**n/N (%)**	**missing data**
Age (years)	50 (29-67)		0
Female		5/15 (33.3)	0
Male		10/15 (66.7)	0

Data are presented as mean (standard deviation) if the Shapiro-Wilk normality test was passed. Alternatively, the median (range) is reported. For nominal data, n/N (%) is reported, where N is the total number of participants with available data. The ICU length is reported for patients admitted to ICU only. Extracorporeal CO2 elimination was performed using the advanced multi-organ support (ADVOS) from Advitos. Renal replacement indicates dialysis systems other than ADVOS.

COPD, chronic obstructive pulmonary disease; ICU, Intensive Care Unit; NIV, non-invasive ventilation; SD, standard deviation; WHO, world health organization.

Supplementary Figure 5: Influence of COVID-19 severity on leukocyte-platelet (LP) aggregate formation

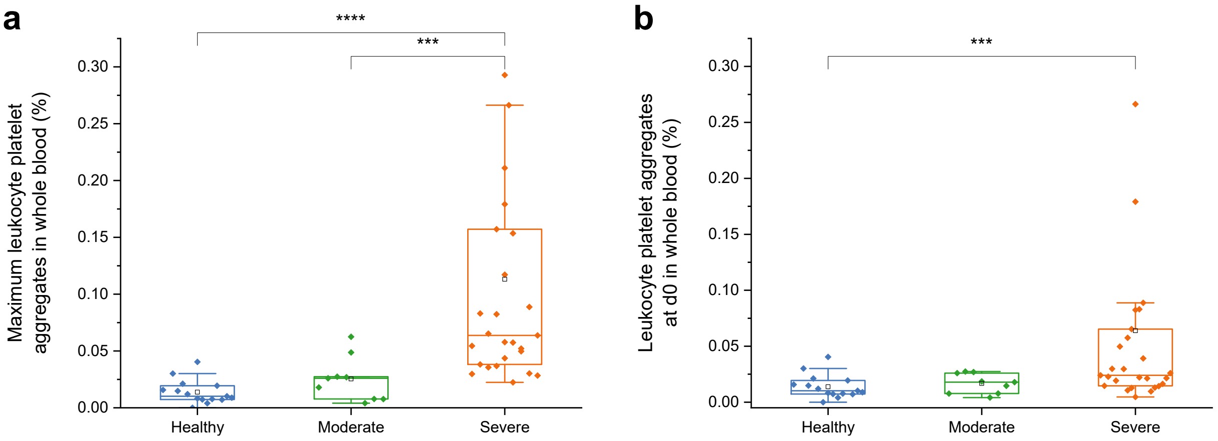

The distribution of LP aggregates is visualised by boxplots for healthy references (n = 15), moderate (n = 9), and severe (n = 27) COVID-19 patients. Each data point represents one patient. The centreline of the boxplots represents the median, and the framed black square is the mean of each distribution. The bottom and top hinges correspond to the first and third quartiles. Significance was tested by a two-sided Kruskal-Wallis ANOVA with Dunn's test and is indicated by asterisks.

a, Comparing the maximum LP aggregates measured during the study period, a significant difference can be observed between healthy vs. severe ($p < 0.0001$) and moderate vs. severe ($p = 0.0004$). **b,** At day 0, LP aggregates are significantly higher in severe COVID-19 patients compared to healthy references (severe vs. healthy, $p = 0.0009$).

Supplementary Figure 6: Additional examples for the size distribution of aggregates in patients with different COVID-19 severity levels

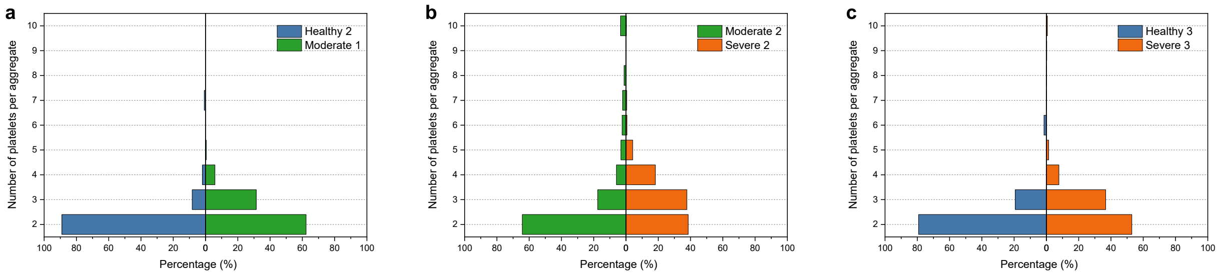

a-c, Additional exemplary size distribution of platelet aggregates for healthy donors (blue), moderate (green), and severe COVID-19 patients (orange). The bar graphs depict the percentage of aggregates with a specific number of platelets.

Supplementary Figure 7: Size of platelets occurring as single cells and in aggregates

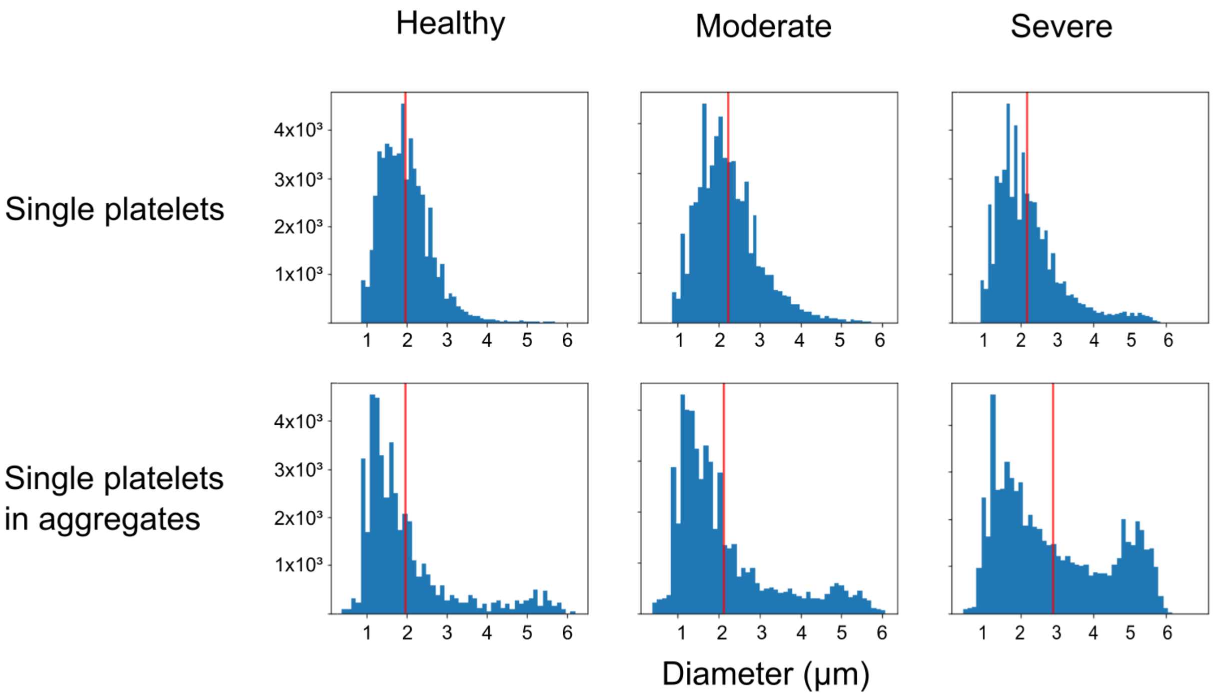

The histograms show the distribution of cell diameters for single platelets (top row) and platelets in aggregates (bottom row) for the healthy (left panel), moderate (middle panel), and severe (right panel) cohorts. While the mean (red line) platelet diameter is approximately 2 µm, a population of larger platelets ranging from 4-6 µm can be

observed. The proportion of larger platelets is higher in PP aggregates and increases with COVID-19 severity.

Supplementary Figure 8: Non-interfering variables

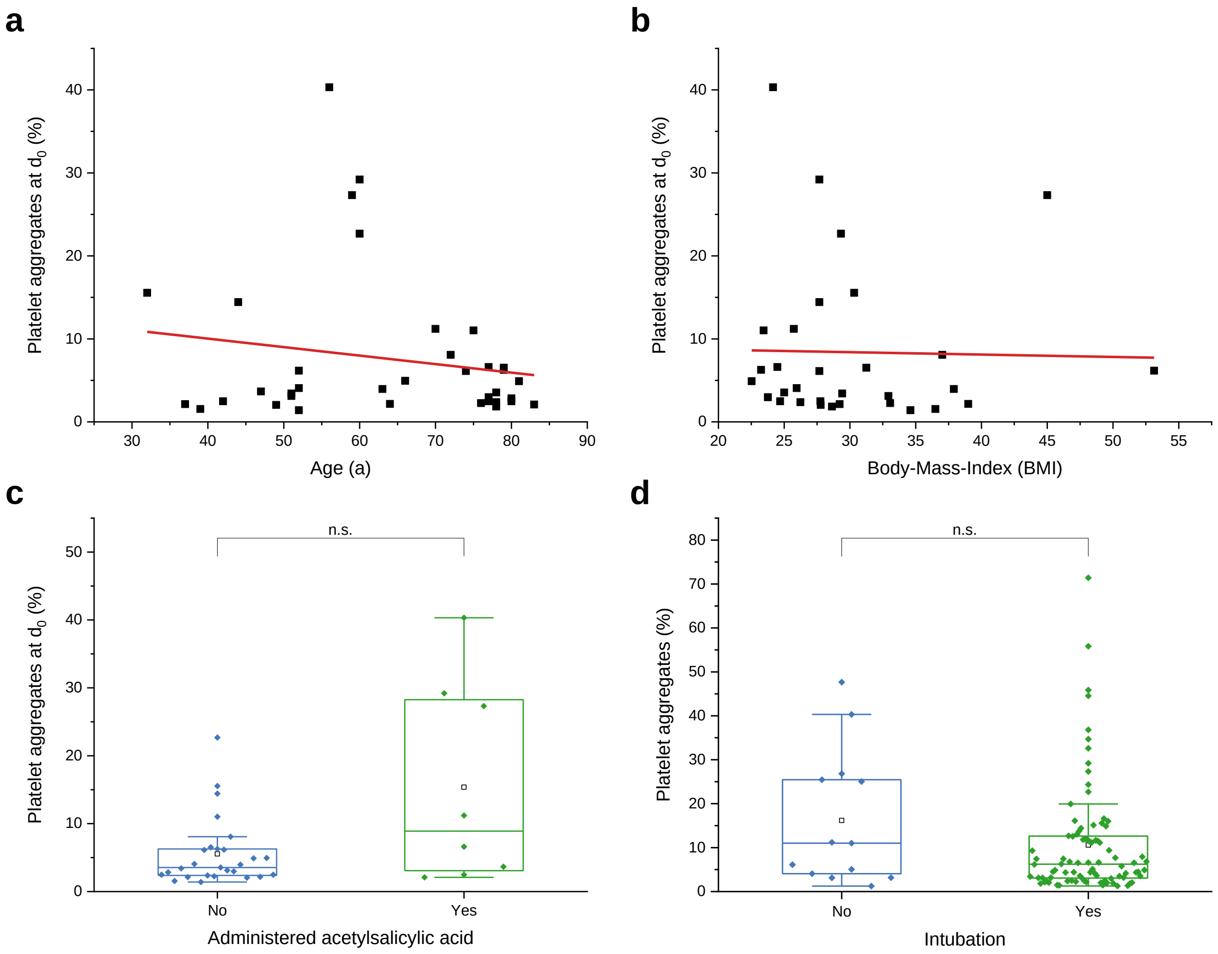

Display of further possible confounding variables like the patient's age (**a**), Body-Mass-Index (BMI, **b**), administration of Aspirin (**c**), and intubation (**d**). **a,** No significant correlation between the age of patients in the cohort and the measured amount of platelet aggregates at d_0 could be observed (r = -0.05, p = 0.7523). **b,** Body-Mass-Index against the measured amount of platelet aggregates at d_0 showed no significant correlation (r = -0.20, p = 0.2815). **c,** Comparison of the PP aggregates (in percentage at day 0) between all COVID-19 patients who were treated with acetylsalicylic acid and all patients who were not. No significant difference was observed (p = 0.0771). **d,**

Distribution of PP aggregates for severe patients, both intubated and not intubated, are visualised by box plots. Each dot represents one measurement, whereby multiple measurements per patient were possible. No significant correlation was found between intubation and the number of aggregates measured ($p = 0.2456$).
