# Supplementary material for: Platelet aggregates detected using quantitative phase imaging associate with COVID-19 severity
Source: Commun Med (Lond). 2023 Nov 7;3:161. doi: 10.1038/s43856-023-00395-6 (PMC10630365; doi:10.1038/s43856-023-00395-6)
Supplement: Supplementary file 3 — Description of Additional Supplementary Files [file 43856_2023_395_MOESM3_ESM.pdf]

## Description of Additional Supplementary Files

**File name:** Supplementary Data 1
**Description:** Values of several established biomarkers for individual patients. These include, among others, Creatinine, Bilirubin, CRP, hs-Troponin T, IL-6, D-Dimer and Procalcitonin.

**File name:** Supplementary Data 2
**Description:** All source data of the main figures.
