# Supplementary material for: Platelet aggregates detected using quantitative phase imaging associate with COVID-19 severity
Source: Commun Med (Lond). 2023 Nov 7;3:161. doi: 10.1038/s43856-023-00395-6 (PMC10630365; doi:10.1038/s43856-023-00395-6)
Supplement: Supplementary file 6 — Reporting Summary [file 43856_2023_395_MOESM6_ESM.pdf]

nature portfolio

Corresponding author(s): Oliver Hayden

Last updated by author(s): Jul 10, 2023

Reporting Summary

Nature Portfolio wishes to improve the reproducibility of the work that we publish. This form provides structure for consistency and transparency in reporting. For further information on Nature Portfolio policies, see our Editorial Policies and the Editorial Policy Checklist.

Statistics

For all statistical analyses, confirm that the following items are present in the figure legend, table legend, main text, or Methods section.

n/a	Confirmed	
☐	☒	The exact sample size (*n*) for each experimental group/condition, given as a discrete number and unit of measurement
☐	☒	A statement on whether measurements were taken from distinct samples or whether the same sample was measured repeatedly
☐	☒	The statistical test(s) used AND whether they are one- or two-sided<br>*Only common tests should be described solely by name; describe more complex techniques in the Methods section.*
☐	☒	A description of all covariates tested
☐	☒	A description of any assumptions or corrections, such as tests of normality and adjustment for multiple comparisons
☐	☒	A full description of the statistical parameters including central tendency (e.g. means) or other basic estimates (e.g. regression coefficient) AND variation (e.g. standard deviation) or associated estimates of uncertainty (e.g. confidence intervals)
☐	☒	For null hypothesis testing, the test statistic (e.g. *F*, *t*, *r*) with confidence intervals, effect sizes, degrees of freedom and *P* value noted<br>*Give P values as exact values whenever suitable.*
☒	☐	For Bayesian analysis, information on the choice of priors and Markov chain Monte Carlo settings
☒	☐	For hierarchical and complex designs, identification of the appropriate level for tests and full reporting of outcomes
☐	☒	Estimates of effect sizes (e.g. Cohen's *d*, Pearson's *r*), indicating how they were calculated

Our web collection on statistics for biologists contains articles on many of the points above.

Software and code

Policy information about availability of computer code

Data collection	OsOne (Ovizio Imaging Systems) - microscope software
Data analysis	IBM SPSS Statistics 25 (SPSS Inc), R version 4.0.2 (R Foundation for statistical computing), Excel version 16.51 (Microsoft), OriginPro 2021b (OriginLab)

For manuscripts utilizing custom algorithms or software that are central to the research but not yet described in published literature, software must be made available to editors and reviewers. We strongly encourage code deposition in a community repository (e.g. GitHub). See the Nature Portfolio guidelines for submitting code & software for further information.

Data

Policy information about availability of data

All manuscripts must include a data availability statement. This statement should provide the following information, where applicable:

- Accession codes, unique identifiers, or web links for publicly available datasets
- A description of any restrictions on data availability
- For clinical datasets or third party data, please ensure that the statement adheres to our policy

Anonymised aggregated data will be available to researchers upon request, which should be directed to the corresponding author.

Research involving human participants, their data, or biological material

Policy information about studies with human participants or human data. See also policy information about sex, gender (identity/presentation), and sexual orientation and race, ethnicity and racism.

Reporting on sex and gender	Sex and gender were not considered in the study design. Sex was assigned according to the medical chart for COVID-19 patients and self-reported for the control cohort. Sex-based analyses were not performed due to a limited sample size. See population characteristics for sex distribution.
Reporting on race, ethnicity, or other socially relevant groupings	Race, ethnicity, or other socially relevant groupings were not considered in the study design.
Population characteristics	COVID-19: median age 65 years (32-83); 33.3% female, 66.7% male; median WHO clinical progression score 9 (4-10). Control cohort: 33.3% female, 66.7 % male; median age 50 (29-67)
Recruitment	Patients with PCR-confirmed SARS-CoV-2-infection admitted to the Klinikum München rechts der Isar were prospectively enrolled and informed consent was obtained from the subjects or their legal representatives.
Ethics oversight	The study was conducted in accordance with the declaration of Helsinki and approved by the ethics committee of the Technical University Hospital of Munich (221/20 S-SR and 620/21 S-KK). Informed consent was obtained from all subjects or their legal representatives.

Note that full information on the approval of the study protocol must also be provided in the manuscript.

Field-specific reporting

Please select the one below that is the best fit for your research. If you are not sure, read the appropriate sections before making your selection.

☒ Life sciences ☐ Behavioural & social sciences ☐ Ecological, evolutionary & environmental sciences

For a reference copy of the document with all sections, see nature.com/documents/nr-reporting-summary-flat.pdf

Life sciences study design

All studies must disclose on these points even when the disclosure is negative.

Sample size	Sample-size calculation was not performed. All patients suitable for the study were recruited.
Data exclusions	Data exclusion was not performed.
Replication	Technical replicates were not performed to reduce aging effects.
Randomization	Patients were allocated to mild/moderate/severe COVID-19 according to the WHO ordinal scale for clinical improvement for hospitalised patients with COVID-19
Blinding	Patient data is only available to the respective medical doctor. For the measurement and evaluation of the data, pseudonymised data was used.

Reporting for specific materials, systems and methods

We require information from authors about some types of materials, experimental systems and methods used in many studies. Here, indicate whether each material, system or method listed is relevant to your study. If you are not sure if a list item applies to your research, read the appropriate section before selecting a response.

Materials & experimental systems

n/a	Involved in the study	
☒	☐	Antibodies
☒	☐	Eukaryotic cell lines
☒	☐	Palaeontology and archaeology
☒	☐	Animals and other organisms
☒	☐	Clinical data
☒	☐	Dual use research of concern
☒	☐	Plants

Methods

n/a	Involved in the study	
☒	☐	ChIP-seq
☒	☐	Flow cytometry
☒	☐	MRI-based neuroimaging
